# Inelastic light scattering by 2D electron system with SO interaction

**DOI:** 10.1186/1556-276X-7-537

**Published:** 2012-09-28

**Authors:** Alexander V Chaplik, Lev I Magarill, Ritta Z Vitlina

**Affiliations:** 1 Institute of Semiconductor Physics, Siberian Branch of the Russian Academy of Sciences, Novosibirsk, 630090, Russia; 2 Novosibirsk State University, Novosibirsk, 630090, Russia

**Keywords:** Two-dimensional system, Inelastic light scattering, Spin-orbit interaction, Rashba model

## Abstract

Inelastic light scattering by electrons of a two-dimensional system taking into account the Rashba spin-orbit interaction (SOI) in the conduction band is theoretically investigated. The case of resonance scattering (frequencies of incident and scattered light are close to the effective distance between conduction and spin-split-off bands of the A_III_B_V_-type semiconductor) is considered. As opposed to the case of SOI absence, the plasmon peak in the scattering occurs even at strictly perpendicular polarizations of the incident and scattered waves. Under definite geometry, one can observe the spectrum features conditioned by only single-particle transitions. In the general case of elliptically polarized incident and scattered light, the amplitude of the plasmon peak turns out to be sensitive to the sign of the SOI coupling.

## Background

It is well known that the spectrum of light scattering by a two-dimensional (2D) electron system is characterized by two contributions. One of them is determined by charge density excitations which is commonly called screened scattering. The shift of frequency equals the 2D plasmon frequency. The maximum of intensity of the corresponding peak in light scattering is reached when polarizations are parallel, and it is equal to 0 when polarizations are perpendicular.

The other contribution corresponds to single-particle excitations (SPE). The typical frequency shift is of the order *qv*_*F*_, where *q* is the wave vector transfer and *v*_*F *_is the Fermi velocity. The intensity of this peak is maximal for perpendicular polarizations of the incident and scattered waves. As to polarized scattering, the SPE contribution strongly depends on the resonance parameter (see [1]): if the incident frequency is close to the effective bandgap (including the Moss-Burstein shift), the SPE peak can be comparable with the plasmon one.

The SOI substantially changes the spectrum of inelastic light scattering. A new peak (of a nontrivial shape) appears with the frequency shift equal to the spin splitting at the Fermi momentum. The polarization dependences are changed qualitatively. The plasmon peak can occur even at crossed polarizations. Finally, the left to right symmetry of circularly polarized incident light is violated: the cross section is invariant under simultaneous change of signs of polarizations and the SOI constant. This allows, in principle, to determine the sign of the Rashba constant experimentally.

## Methods

### Expressions for the scattering cross section

In the random-phase approximation, the differential cross section for the scattering by a 2D system can be written as follows [2-4]:

(1) $$\frac{d^{2}\sigma }{d\omega d\Omega }=\frac{\omega_{2}}{\omega_{1}}{\left(\frac{e}{c}\right)}^{4}\frac{n_{\omega }+1}{\Pi }\text{Im}\left(L_{2}-\frac{2\Pi e^{2}}{q\kappa }\frac{L_{1}{\tilde{L}}_{1}}{\varepsilon }\right),$$

where *L*_2_,*L*_1_, and ${\tilde{L}}_{1}$ are respectively given by the expressions

(2) $$\begin{array}{ll} L_{2}=\frac{1}{S}\underset{\beta^{\prime }\beta }{\sum }|\gamma_{\beta^{\prime }\beta }{|}^{2}F_{\beta^{\prime }\beta }, & L_{1}=\frac{1}{S}\underset{\beta^{\prime }\beta }{\sum }\gamma_{\beta^{\prime }\beta }^{*}J_{\beta^{\prime }\beta }(q)F_{\beta^{\prime }\beta },\;\\ & {\tilde{L}}_{1}=\frac{1}{S}\underset{\beta^{\prime }\beta }{\sum }\gamma_{\beta^{\prime }\beta }J_{\beta^{\prime }\beta }^{*}(q)F_{\beta^{\prime }\beta }.\;\;\;\; \end{array}$$

Here, *ω*_1,2_ are the incident and scattered light frequencies, respectively; **q **=** q**_1 _−** q**_2_, **q**_1,2 _are the in-plane components of the incident and scattered light wave vectors, respectively; *ω *=* ω*_1_−*ω*_2_ is the frequency shift in the inelastic light scattering; *n*_*ω *_= 1/(*e*^*ω*/*T*^−1) is the Bose distribution function; *J*(**q**) =* e*^*i***qr**^, *β* is the set of quantum numbers characterizing an electron state in the conduction band; *S* is the normalization area; *κ* is the background dielectric constant; $\hat{\gamma }$ is the scattering operator; and *ℏ *= 1 is assumed throughout this paper. The longitudinal dielectric function of electrons in the conduction band *ε* has the form

(3) $$\varepsilon (\omega ,q)=1+\frac{2\Pi e^{2}}{q\kappa }\frac{1}{S}\underset{\beta^{\prime }\beta }{\sum }|J_{\beta^{\prime }\beta }(q){|}^{2}F_{\beta^{\prime }\beta },$$

(4) $$F_{\beta^{\prime }\beta }=\frac{f_{\beta^{\prime }}-f_{\beta }}{\left(\omega +\varepsilon_{\beta \beta^{\prime }}+i\delta \right)},\;(\delta =+0),$$

where *f*_*β *_≡* f*(*ε*_*β*_), *f*(*ε*) is the Fermi distribution function, *ε*_*β*_ is the energy of an electron in the conduction band, and $\varepsilon_{\beta \beta^{\prime }}=\varepsilon_{\beta }-\varepsilon_{\beta^{\prime }}$.

The resonant situation is considered when the frequencies of incident (scattered) wave *ω*_1_(*ω*_2_) are close to *E*_0_ + *Δ*_0_, i.e., resonance with the spin-orbit split-off band takes place (*E*_0_ and *Δ*_0_ are the band parameters of the bulk A_III_B_V_ semiconductor). In this case, the operator of scattering $\hat{\gamma }$ reads

(5) $$\hat{\gamma }=\hat{\gamma_{1}}+\hat{\gamma_{2}}=A((e_{1}e_{2}^{*})+i(\sigma a))J(q),\;A=\frac{1}{3}\frac{P^{2}}{E_{g}-\omega_{1}},$$

where *E*_*g*_ is the effective bandgap width, $a=[e_{1},e_{2}^{*}]$, *P *≡* p*_*cv*_/*m*_0_ is the Kane parameter, **e**_1,2_ are the polarizations of incident and scattered photons, and ***σ ***are the Pauli matrices. We treat here the enhanced resonant factor *A* in Equation 5 just as a constant that is true for *not* extremely resonant regime: the denominator in Equation 5 is much larger than the Fermi energy of electrons. We do this in order to simplify calculations because our main goal in this paper is to demonstrate the qualitatively new features of the scattering process due to spin-orbit interaction.

The substitution of Equation 5 into Equation 1 yields an expression comprising four characteristic contributions to the scattering:

(6) $$\frac{d^{2}\sigma }{d\omega d\Omega }=\frac{\omega_{2}}{\omega_{1}}{\left(\frac{e}{c}\right)}^{4}\frac{n_{\omega }+1}{\Pi }R(\omega ),R(\omega )=\overset{4}{\underset{j=1}{\sum }}R_{j}(\omega ),$$

where

(7) $$R_{1}(\omega )=-\frac{A^{2}\kappa q}{2\Pi e^{2}}|e_{1}\cdot e_{2}^{*}{|}^{2}\text{Im}\left(\frac{1}{\varepsilon }\right),$$

(8) $$R_{2}(\omega )=\frac{1}{S}\underset{\beta^{\prime }\beta }{\sum }|{\left(\gamma_{2}\right)}_{\beta^{\prime }\beta }{|}^{2}\text{Im}(F_{\beta^{\prime }\beta }),$$

(9) $$R_{3}(\omega )=-\frac{2\Pi e^{2}}{q\kappa }\text{Im}\left(\frac{Z\tilde{Z}}{\varepsilon }\right),$$

(10) $$R_{4}(\omega )=A\;\text{Im}\left(\frac{1}{\varepsilon }\left((e_{1}e_{2}^{*})Z+(e_{1}^{*}e_{2})\tilde{Z}\right)\right).$$

The values of *Z* and $\tilde{Z}$ are given by expressions for *L*_1 _and ${\tilde{L}}_{1}$ in Equation 2 with *γ *replaced by *γ*_2_.

The contribution *R*_1 _determines the scattering of light by fluctuations of charge density. The value *R*_2 _determines unscreened mechanism of scattering and corresponds to single-particle excitations. It can be shown that in the absence of SOI in the conduction band, the values of *Z* and $\tilde{Z}$ and, respectively, *R*_3 _and *R*_4_ are equal to 0 identically.

Equations 7 to 10 are general. They are valid for any Hamiltonian, describing electron states in the conduction band. In this paper, we consider the light scattering for the so-called Rashba plane, namely 2D electron gas in the presence of SOI. Such a system is described by the Hamiltonian [5]

(11) $${ℋ}_{0}=\frac{p^{2}}{2m}+\alpha (\sigma \cdot [p,n]).$$

Here, **p** is the 2D momentum of the electron, *m* is the effective mass, *α *is the Rashba parameter, and **n**is the unit vector normal to the plane of the system. The spectrum of this Hamiltonian has the form

(12) $$\varepsilon_{\beta }=p^{2}/2m+\mu \alpha p,$$

where *β *= (**p**,*μ*) and the parameter *μ *= ± 1 labels two branches of the spin-split spectrum. The wave functions of the Hamiltonian (Equation 11) are

(13) $$\psi_{p,\mu }=\frac{e^{i\mathrm{pr}}}{\sqrt{2S}}\left(\begin{array}{c} i\mu e^{-i\varphi_{p}}\\ \;\;\;\;1 \end{array}\right).$$

## Results and discussion

### Numerical calculations

**Figure 1:**
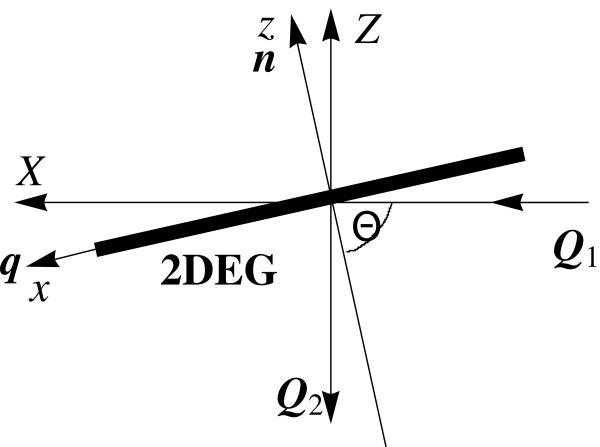
**Scattering configuration for which numerical calculations are performed.** The incident (**Q**_**1**_) and scattered (**Q**_**2**_) beams form a right angle; *θ *is the angle of incidence.

Equations 7 to 10, 12, and 13 were used for numerical calculations of scattering cross section as a function of frequency shift *ω*. They were carried out for 2D electron gas at temperature *T *= 0 in the scattering geometry when incident and scattered beams make a right angle and lie in the same plane (Figure 1). The structure InAs/GaSb with *α *= 1.44 × 10^6 ^cm/s, *m *= 0.055 *m*_0_, and *κ *= 15.69 was considered at the areal concentration *n*_*s*_= 10^11 ^cm^−2^. The contributions *R*_1_,*R*_3_, and *R*_4_ contain plasmon poles (zeros of *ε*). To get finite results, it is necessary to introduce a finite damping. We replace *δ* in Equation 4 by the relaxation frequency *ν *=* e*/*mμ* (*μ* is the mobility). For *q* and *ν*, we have chosen the following values: *ℏq *= 0.004 *p*_*F*_,* ℏν *= 0.001* ε*_*F*_.

**Figure 2:**
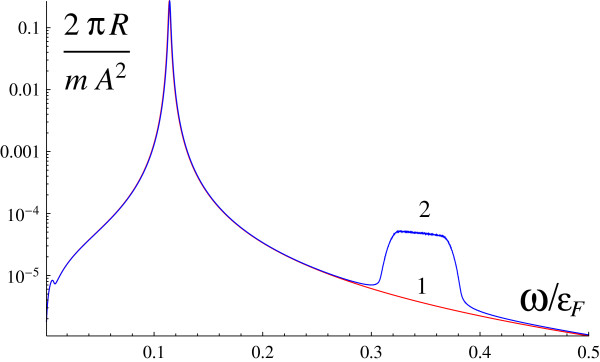
**Scattering cross section versus frequency shift fore**_**1**_**∥e**_**2 **_**.***α *= 0 (1, red line); *α *= 1.44 × 10^6 ^cm/s (2, blue line).

If the polarizations of incident and scattered waves are strictly parallel (**e**_1_||**e**_2_), the cross section is determined by only *R*_1_. The spectrum of scattering has two peaks: the plasmon peak $\omega_{0}(q)=v_{F}\sqrt{q/a_{B}}$ and the SOI-induced peak at 2*αp*_*F*_. The dependence of the cross section on the frequency shift for both peaks is similar to the plasmon absorption and is given by Figure 2.

**Figure 3:**
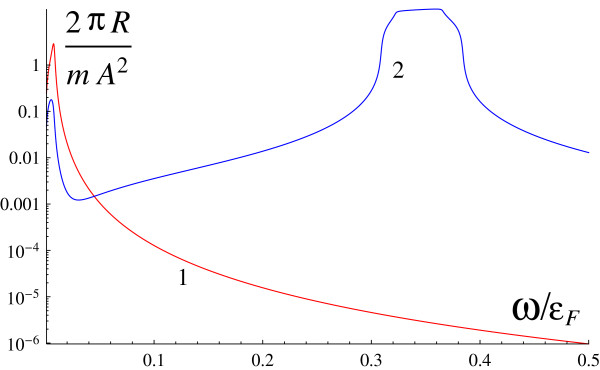
**Scattering cross section versus frequency shift for *α *= 0 (1, red) and *α *= 1.44 ×10**^**6**^**cm/s (2, blue).** The angle of incidence is ***θ *= *Π*/6**.

Let the geometry of scattering in such a way that incident and scattered waves are linearly polarized and, moreover, **e**_1 _⊥** e**_2_ and *a*_*y *_= 0. It can be realized, e.g., if incident and scattered beams are perpendicular and one of them is polarized in the incidence plane but the other is perpendicular to it. In this case, the spectrum demonstrates peculiarities due to only single-particle transitions (contribution *R*_2_): one peak near the frequency *qv*_*F*_ and another peak near the frequency 2*αp*_*F*_. This case is demonstrated by Figure 3.

**Figure 4:**
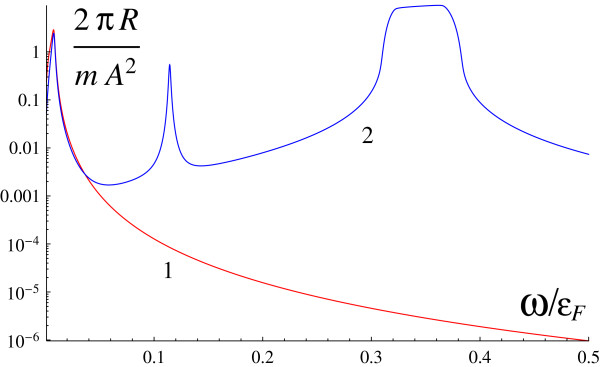
**Scattering cross section versus frequency shift for *α *** **= 0(1, red) and *α *** **= 1.44 ×10**^**6**^**cm/s (2, blue).**

Due to SOI, the plasmon peak in the light scattering spectrum can occur even at strictly perpendicular polarizations of incident and scattered waves. It occurs when vector **a **has a nonzero projection onto axis *y* (axes *z* and *x* were chosen along vectors **n** and **q**, respectively). The nonvanishing contribution to the cross section is due to the sum *R*_2_ + *R*_3_. This case is presented by Figure 4.

**Figure 5:**
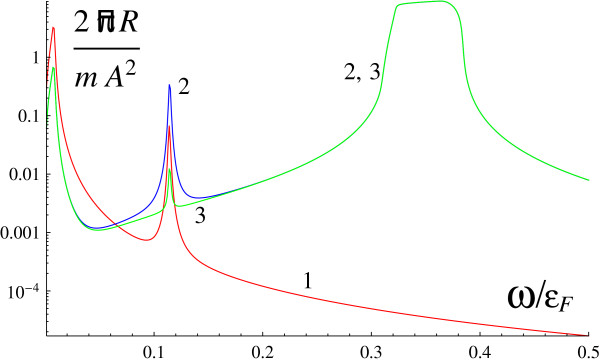
**Dependence of scattering cross section on frequency when one of the waves has circular polarization.***α *= 0 (1, red), *α *= 1.44 × 10^6^cm/s (2, blue and 3, green); incident wave with left polarization (2, blue), incident wave with right polarization (3, green).

For the existence of the contribution *R*_4_, polarization vectors **e**_1 _and **e**_2 _should be arbitrarily oriented with respect to each other (neither parallel nor perpendicular). Besides, at least one of the waves should not be linearly polarized. When these conditions are justified and *R*_4 _≠ 0, all other contributions (*R*_1_,*R*_2_,*R*_3_) also exist. Herewith, due to the sensitivity of the contribution *R*_4_ to the sign of the effective Rashba SOI *α*and to polarization vector phases, the total cross section of scattering also depends on these parameters. Therefore, measurements of inelastic light scattering can be, in principle, used for the determination of the sign of the constant *α*. Figure 5 shows an example of the inelastic light scattering spectrum in the most interesting case when the incident wave has right or left circular polarization while the scattered one is linearly polarized at the angle *Π*/4 to the incidence plane. It is seen that at *α *> 0, the amplitude of the plasmon peak for left polarization is distinctly larger than that for right polarization. For *α *< 0, the curves should be interchanged.

## Conclusions

Thus, allowing SOI essentially (qualitatively) changes the spectrum of inelastic light scattering by a 2D electron system. It should be especially noted that in the absence of external magnetic field, the symmetry between left and right polarizations is violated.

## Competing interests

The authors declare that they have no competing interests.

## Authors’ contributions

AVC, LIM, and RZV equally contributed in writing the manuscript and in performing the theoretical analysis. All authors read and approved the final manuscript.
